# Impact of age on outcome of CAR-T cell therapies for large B-cell lymphoma: the GLA/DRST experience

**DOI:** 10.1038/s41409-022-01867-4

**Published:** 2022-11-22

**Authors:** Peter Dreger, Udo Holtick, Marion Subklewe, Bastian von Tresckow, Francis Ayuk, Eva Wagner, Gerald Wulf, Reinhardt Marks, Olaf Penack, Ulf Schnetzke, Christian Koenecke, Malte von Bonin, Matthias Stelljes, Bertram Glass, Claudia D. Baldus, Vladan Vucinic, Dimitrios Mougiakakos, Max Topp, Roland Schroers, Daniel Wolff, Simone Thomas, Nicolaus Kröger, Wolfgang A. Bethge, on behalf of the German Lymphoma Alliance (GLA), the German Stem Cell Transplantation Registry (DRST)

**Affiliations:** 1 University Hospital Heidelberg, Department of Hematology & Oncology, Heidelberg, Germany; 2 Department I of Internal Medicine, Medical Faculty and University Hospital of Cologne, University of Cologne, Cologne, Germany; 3 University Hospital Munich (LMU Munich), Department of Hematology & Oncology, Munich, Germany; 4 Department of Hematology and Stem Cell Transplantation, West German Cancer Center, University Hospital Essen, University of Duisburg-Essen, Essen, Germany; 5 University Hospital Hamburg, Department for Stem Cell Transplantation, Hamburg, Germany; 6 University Hospital Mainz, Department of Hematology & Oncology, Mainz, Germany; 7 University Medicine Goettingen, Clinic for Hematology & Medical Oncology, Göttingen, Germany; 8 University Hospital Freiburg, Department of Hematology & Oncology, Freiburg, Germany; 9 University Hospital Charite, Department of Hematology & Oncology, Berlin, Germany; 10 University Hospital Jena, Department of Hematology & Oncology, Jena, Germany; 11 University Hospital Hannover, Department of Hematology & Oncology, Hannover, Germany; 12 University Hospital Dresden, Department of Hematology & Oncology, Dresden, Germany; 13 University Hospital Muenster, Department of Hematology & Oncology, Muenste, Germany; 14 Klinikum Berlin-Buch, Department of Hematology & Oncology, Berlin, Germany; 15 University Hospital Kiel, Department of Hematology & Oncology, Kiel, Germany; 16 University Hospital Leipzig, Leipzig, Germany; 17 University Hospital Erlangen, Department of Hematology & Oncology, Erlangen, currently University Hospital Magdeburg, Department of Hematology, Magdeburg, Germany; 18 University Hospital Würzburg, Department of Hematology & Oncology, Würzburg, Germany; 19 University Hospital Bochum, Department of Hematology & Oncology, Bochum, Germany; 20 University Hospital Regensburg, Department ofInternal Medicine III, Hematology and Oncology, Regensburg, Germany; 21 Leibniz Institute for Immunotherapy, Regensburg, Germany; 22 University Hospital Tuebingen, Department of Hematology & Oncology, Tuebingen, Germany

**Keywords:** Immunotherapy, Disease-free survival

## To the Editor:

Treatment with CD19-directed CAR T-cells has evolved as a standard of care for relapsed or refractory large B-cell lymphoma (r/r LBCL). As LBCL is largely a disease of the elderly, age limitations of CAR T-cell therapy may affect its applicability. Notably, age has not been among the unfavorable predictors of progression-free survival (PFS) in our recent analysis of commercial use of axicabtagene ciloleucel (axi-cel) and tisagenlecleucel (tisa-cel) in Germany [1]. Actually, the hazard ratio of 0.904 (0.825–0.990) per decade suggested that the outcome of CAR-T treatment *improved* with increasing age. In order to have a closer look on this remarkable finding, we conducted a follow-up analysis comparing patients younger and older than 65 years in our sample.

Between November 2018 and April 2021 356 patients received axi-cel (*n* = 173) or tisa-cel (*n* = 183) for standard-of-care (SOC) treatment of r/r LBCL. Of these, 140 patients were aged 65 years or older (median age 71 years, range 65–83), whereas the remainder was younger than 65 years (median 53 years, range 19–64). There were no significant differences between the two age cohorts in terms of gender, LBCL subset, pretreatment lines, performance status, LDH at lymphodepletion, International Prognostic Index, ZUMA-1 eligibility, and CAR product used. However, the younger group had a significantly shorter time from diagnosis to dosing, contained a significantly higher proportion of patients who had failed hematopoietic cell transplantation, and tended to have a smaller fraction of bridging responders (Supplementary Table S1). The median follow-up was 11 months.

Regarding toxicities, older and younger patients did not differ in terms of duration of hospitalization and incidence of higher-grade cytokine release syndrome. However, patients aged ≥65 years had an almost doubled risk of higher-grade neurotoxicity, both with axi-cel and tisa-cel, even though this was not statistically significant (Supplementary Table S2). Furthermore, non-relapse mortality (NRM) tended to be higher in the elderly group: 12-month cumulative NRM incidence considering relapse/progression as competing risks was 9% (95% confidence interval (95% CI) 4–14%) in patients ≥65 years vs 3% (95% CI 1–5%) in patients <65 years; harzard ratio (HR) 2.25 (95% CI 0.93–5.43). This effect was similar across the two products used, taking into account that overall NRM was significantly lower with tisa-cel compared to axi-cel [1] (Supplementary Fig. S1). In both age cohorts, infections were the leading cause of non-relapse death accounting for two thirds of the events in each group.

**Fig. 1:**
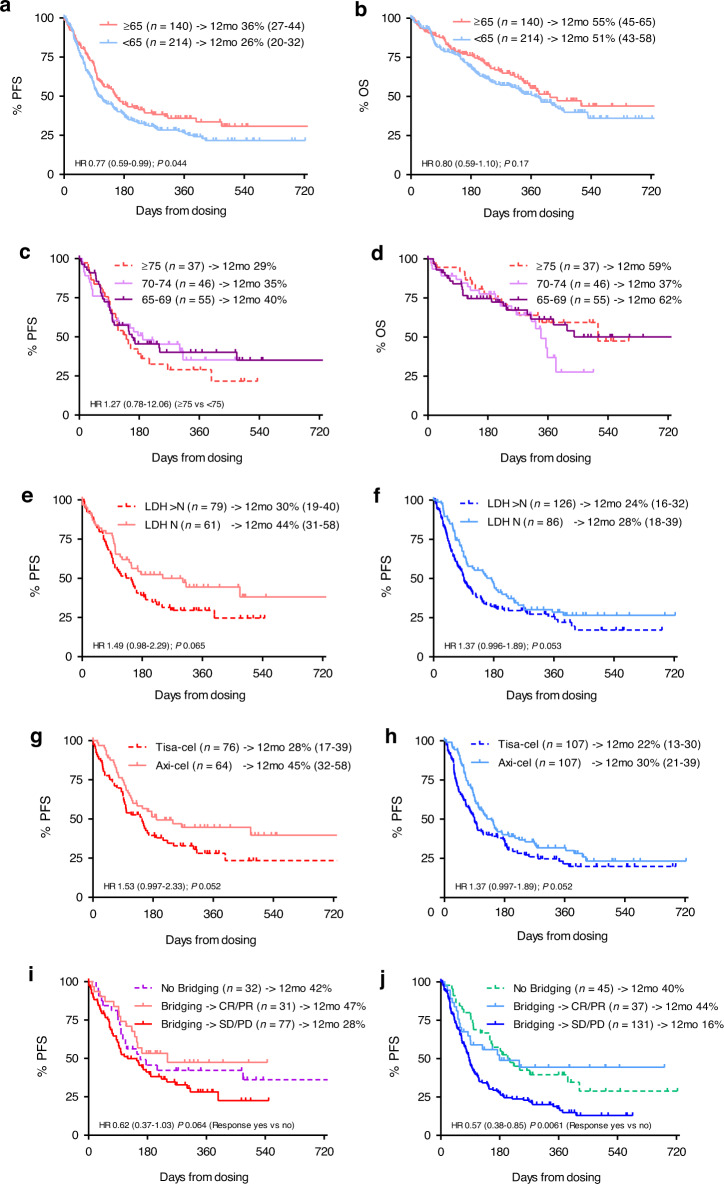
Survival outcomes by age. PFS (**a**) and OS (**b**) of patients ≥65 or <65 years; PFS (**c**) and OS (**d**) of patients aged 65–69, 70–74, and ≥75 years; age effects on PFS by LDH (**e**, **f**), CAR product used (**g**, **h**), and bridging (**i**, **j**) (left panels **e**, **g**, **i** ≥ 65 years; right panels **f**, **h**, **j** < 65 years). Comparisons were made by Log-rank tests.

With 69 and 43%, respectively, overall response rate (ORR) and rate of complete responses (CR) were significantly higher in patients aged ≥ 65 years than in younger patients (58 and 31%, respectively, Supplementary Table S2). Also the response benefit of the elderly was observed across both products, but it appeared more pronounced with axi-cel compared to tisa-cel (ORR 89 and 70% in older and younger patients, respectively, with axi-cel, *p* = 0.0073; vs 61 and 50%, respectively, with tisa-cel, *p* = 0.16; CR 56 and 35% with axi-cel, *p* = 0.0094; and 37 and 29% with tisa-cel, *p* = 0.33). The superior response rates in the elderly translated into a significantly better progression-free survival (PFS, at 12 months 36 and 26% for patients aged ≥65 years and <65 years, respectively), whereas overall survival was not significantly different (Fig. 1a, b). When subdividing the elderly cohort further into age groups 65–69, 70–74, and ≥75 years, we did not observe significant survival differences between the age categories (Fig. 1c, d).

Apart from age, risk factors for PFS in the total sample were product used, LDH, and bridging [1]. The beneficial effects of normal LDH and axi-cel appeared to be even more pronounced in the upper age group than in younger patients, including the subset being 75 years or older (Supplementary Fig. S2). In contrast, the impact of bridging and the response to it was less impressive in patients aged ≥65 years compared to those < 65 years (Fig. 1e–j). This was due to the fact that the PFS of younger patients who did not respond to bridging was particularly poor (12-month PFS only 16% (95% CI 9–23%).

There have been a few studies suggesting that advanced age is not a major obstacle for CD19-directed CAR-T therapy in r/r LBCL [2–5], including the recent large post-authorization safety study conducted by the CIBMTR for axi-cel [6]. In the latter, ORR and PFS tended to be better in 484 patients aged 65 or higher compared to 813 younger patients on multivariate analysis, despite significantly higher risks of overall and grade ≥3 CRS and neurotoxocity, respectively, in the elderly [6]. In contrast, neurotoxities were not found to be significantly increased in older patients in two smaller studies with axi-cel [7] and tisa-cel [8], respectively. Finally, preliminary data from the ZUMA-7 trial, investigating axi-cel as second-line treatment for LBCL, showed a higher CR rate in patients ≥65 years [9, 10].

Since there is no reason to believe that older patients have less aggressive tumors or a more functional T-cell system, the most plausible explanation for the superior efficacy especially of axi-cel in the elderly is patient selection. Although the beneficial age effects remained stable after multivariable adjustment for confounders in our cohort [1] and the CIBMTR study [6], there might be risk factors that are not reflected by the parameters considered in the multivariate analyses. Indeed, although we did not assess the time between start of 1^st^-line therapy and first relapse/progression, the shorter interval between diagnosis and dosing in the younger cohort suggests a larger fraction of patients with primary/early treatment failure, despite more aggressive induction and also salvage therapy (Supplementary Table S2). Thus, the reason for the difference between the age groups observed here might be that our younger patients obviously represent an extraordinarily unfavorable selection [11], while the outcome of the elderly is comparable to other published real-world experience [4–6].

Not surprisingly, the risks of grade ≥3 neurotoxicity and NRM tended to be higher in the elderly cohort, especially after axi-cel treatment. This drawback, however, was overcompensated by the better tumor control provided by axi-cel in the older patient group, with the result that the PFS superiority of axi-cel over tisa-cel was particularly pronounced in the elderly. Taking into account that this effect was stable up to the age group of 75 years or older, this finding contradicts current perceptions considering tisa-cel as the preferred choice for older patients because of its better tolerability [1] and, thus, might have impact on clinical practice.

Of note, detrimental age effects did not emerge in our series even when the elderly group was further sub-categorized, implying that a meaningful upper age limit for CD19 CARTs in this indication could not be defined.

This study is limited by sample size, its retrospective character with inherent selection bias, and the fact that it is a post-hoc analysis. Nevertheless, its results suggest that increasing age per se is not a risk factor for outcome of CD19 CAR-T-cell therapy in LBCL, and a strict upper age limit for this type of treatment does not exist. Finally, despite higher NRM, PFS tended to be better with axi-cel compared to tisa-cel also in the elderly, suggesting that tisa-cel is not the obligatory choice for this subset including selected patients aged 75 years or older.

## Supplementary information

Suppl. material

## Data Availability

All data generated or analyzed during this study are included in this published article [and its supplementary information files].
