# Supplementary material for: Impact of age on outcome of CAR-T cell therapies for large B-cell lymphoma: the GLA/DRST experience
Source: Bone Marrow Transplant. 2022 Nov 22;58(2):229–32. doi: 10.1038/s41409-022-01867-4 (PMC9902271; doi:10.1038/s41409-022-01867-4)
Supplement: Supplementary file 1 — Suppl. material [file 41409_2022_1867_MOESM1_ESM.docx]

**Supplemental Appendix**

**Content**

Table S1: Patient characteristics by age……………………………..………………………………….…………………………………………………..…………….. 2

Table S2: Outcomes by age…………………….………………………..…………………………………….…………………………………………..……..…………….. 3

Table S3: Pretreatment details by age……………………………..…………………………………….…………………………………………………..…………….. 4

Figure S1: Non-relapse mortality by age……………………………………………………………………………………………………………………..…………….. 5

Figure S2: Survival by product used in patients 65-69, 70-74, and ≥75 years old………………………………………………………………………. 6

**Table 1:** Patient characteristics at lymphodepletion by age

|  | **≥65 years (n=140)** | **<65 years**  **(n=216)** | **P value*** |
| --- | --- | --- | --- |
| **Baseline** |  | | |
| Median Age (range) | 71 (65-83) | 53 (19-64) | - |
| Sex male | 94 (67%) | 142 (66%) | 0.82 |
| Diagnosis  DLBCL  Transformed indolent  PMBCL  other | 117 (84%)  21 (15%)  0  2 (1%) | 176 (81%)  22 (10%)  14 (6%)  4 (2%) | 0.65  (DLBCL vs other) |
| **Time from diagnosis (months)** | **20 (4-255)** | **16 (3-310)** | **0.007** |
| # lines failed | 3 (2-7) | 3 (2-9) | 0.078 |
| **Failed HCT** | **38 (27%)** | **83 (38%)** | **0.03** |
| Performance status ≥2 | 24/134 (18%) | 32/208 (15%) | 0.55 |
| LDH >N | 79 (56%) | 129/214 (60%) | 0.51 |
| sIPI high-intermediate/ high ¶ | 78/129 (60%) | 106/201 (53%) | 0.17 |
| ZUMA-1 ineligible | 124 (89%) | 186 (86%) | 0.74 |
| Bridging  None  **Successful (CR/PR)**  Unsuccessful (SD/PD)  No info | 31 (22%)  **29 (21%)**  80 (57%)  0 | 45 (21%)  **29 (13%)**  132 (61%)  10 (5%) | 0.099 vs SD/PD |
| Tisa-cel | 76 (54%) | 107 (50%) | 0.45 |

¶ age-adjusted IPI was applied to patients ≤60 years

* Categorical variables were compared by Fisher’s exact test, and continuous variables by Mann-Whitney test

CR, complete response; HCT, Hematopoietic cell transplantation; ORR, overall response rate; PD, progressive disease; PR, partial response; SD, stable disease; sIPI, secondary International Prognostic Index; N, normal

**Table S2:** Outcomes by age

|  | **≥65 years (n=140)** | **<65 years**  **(n=216)** | **P value*** |
| --- | --- | --- | --- |
| **Outcomes** |  | | |
| Neurotoxicity ≥3  Axi-cel  Tisa-cel | 21/131 (16%)  14/62 (23%)  7/69 (10%) | 18/204 (9%)  13/105 (12%)  5/98 (5%) | 0.055  0.13  0.24 |
| CRS ≥3  Axi-cel  Tisa-cel | 14/136 (10%)  5/63 (8%)  9/73 (12%) | 28/210 (13%)  14/109 (13%)  14/101 (14%) | 0.50 |
| Hospitalization days (median, range) | 22 (8-95) | 21 (9-128) | 0.48 |
| ORR  Axi-cel  Tisa-cel | 97 (69%)  54 (89%)  43 (61%) | 125 (58%)  73 (70%)  52 (50%) | 0.043  0.0073  0.16 |
| CR  Axi-cel  Tisa-cel | 60 (43%)  34 (56%)  26 (37%) | 66 (31%)  36 (35%)  30 (29%) | 0.023  0.0094  0.33 |

* Categorical variables were compared by Fisher’s exact test, and continuous variables by Mann-Whitney test

CR, complete response; CRS, cytokine release syndrome; ORR, overall response rate

**Table S3: Pretreatment details by age (Bridging therapy excluded)**

|  | **≥65 years** | **<65 years** | **p** |
| --- | --- | --- | --- |
| N evaluable | 107 | 160 | - |
| **1^st^ line** |  | | |
| CHOP-like + etoposide ¶ | 5 (5%) | 35 (22%) | <0.0001 |
| **Salvage** |  | | |
| Received >1 standard salvage CIT regimens* | 22 (21%) | 55 (34%) | 0.019 |
| Received any Polatuzumab-containing regimen | 14 (13%) | 14 (9%) | 0.31 |
| **Any line** |  | | |
| Received any Bendamustin-containing regimen | 20 (19%) | 17 (11%) | 0.072 |
| Received radiotherapy | 39 (36%) | 42 (26%) | 0.079 |
| **Interval diagnosis - dosing** |  | | |
| ≤12 months (proportion of patients; %) | 36/136 (26%) | 75/215 (35%) | 0.10 |
| ≤15 months (proportion of patients; %) | 50/136 (37%) | 103/215 (48%) | 0.047 |
| ≤18 months (proportion of patients; %) | 61/136 (45%) | 127/215 (59%) | 0.011 |
| ≤24 months (proportion of patients; %) | 82/136 (60%) | 155/215 (72%) | 0.026 |

¶ R-CHOEP, R-EPOCH

* R-DHAP, R-ICE, R-ESHAP, MATRIX, Dexa-BEAM, R-HD-Mtx/Ifo

**Figure S1:** Non-relapse mortality by age group (≥65 vs <65 years)

**Tisa-cel**

**All**

**Axi-cel**

**Figure S2:** Survival by product used in patients 65-69, 70-74, and ≥75 years old

**A** 65-69 **B** 70-74 **C** ≥75

OS

PFS
